# Neuropsychological assessment of Romanian burning mouth syndrome patients: stress, depression, sleep disturbance, and verbal fluency impairments

**DOI:** 10.3389/fpsyg.2023.1176147

**Published:** 2023-05-15

**Authors:** Cosmin Dugan, Bogdan Ovidiu Popescu, Serban Țovaru, Ioanina Părlătescu, Ioana Andreea Musat, Maria Dobre, Athena Cristina Ribigan, Elena Milanesi

**Affiliations:** ^1^Faculty of Medicine, Carol Davila University of Medicine and Pharmacy, Bucharest, Romania; ^2^Clinical Neurosciences, Geriatrics and Gerontology Departments, Carol Davila University of Medicine and Pharmacy, Bucharest, Romania; ^3^Victor Babeș National Institute of Pathology, Bucharest, Romania; ^4^Department of Oral Pathology, Faculty of Dentistry, Carol Davila University of Medicine and Pharmacy, Bucharest, Romania; ^5^Neurology Department, University Emergency Hospital, Bucharest, Romania

**Keywords:** burning mouth syndrome, stress, depression, sleep disturbance, verbal fluency

## Abstract

Burning Mouth Syndrome (BMS) is a chronic condition characterized by a burning sensation in the oral mucosa, lasting more than 2 hours daily for more than 3 months, without clinical and/or laboratory evidence. BMS is often comorbid with mood, and psychiatric disorders, and a complex pathophysiology and interaction between impairments in nociceptive processing and psychologic function is occurring. In this work, we aimed to define the neuropsychological profile specific for BMS patients for a better management of this complex disease. We conducted a case–control study comparing 120 BMS patients and 110 non-BMS individuals (CTRL). Sociodemographic data and lifestyle habits, were collected, along with data regarding quality of life (SF-36 scale), stress (PSS), depression and anxiety (MADRS and HADS scales), sleep quality (PSQI scale), and cognitive functions (MoCA, SVF and PVF tests). The statistical analysis revealed a lower general quality of life (*p* < 0.001), worse sleep quality (*p* < 0.001) in BMS patients than CTRL. The BMS patients also displayed a higher prevalence of mild depressive symptoms than CTRL applying the MADRS (*p* < 0.001) and HADS-Depression scales (*p* = 0.001), whereas no differences in anxiety symptoms were found between the two groups (*p* = 0.174). Moreover, reduced scores semantic and phonemic verbal fluency tests (*p* < 0.05) were found, but no change in cognition was observed through MoCA (*p* = 0.551). Our results highlight that synergy between dentistry and neuropsychiatric assessment is essential for a successful management of BMS.

## Introduction

Burning mouth syndrome (BMS) is defined by the World Health Organization as a disease of the oral mucosa characterized by persistent chronic pain without clinical lesions and with normal biological investigations (World Health Organization, 2023). BMS is a complex, multifactorial, neuropathic chronic pain mainly described by the patients as a burning sensation or pain (Adamo and Spagnuolo, 2022). The diagnostic criteria stated by the International Headache Society mention that the symptoms are present every day for more than 2 hours, last for more than 3 months, are usually bilateral, and have variable intensity (Headache Classification Committee of the International Headache Society (IHS), 2018). The oral mucosa has a normal clinical aspect. The main symptom features have a large heterogeneity, including “burn,” “hot,” “stinging,” “tingling,”” itching,” “intraoral foreign body sensation,” “altered or unpleasant oral sensation,” and associated symptoms represented by taste disturbances and dry mouth (Chmieliauskaite et al., 2021; Adamo and Spagnuolo, 2022).

A recent meta-analysis reported that the overall pooled prevalence of BMS is 1.73%, with a higher prevalence in females than males (1.15% vs. 0.38%) (Wu et al., 2021). Moreover, the prevalence seems to be increasing in post-menopausal women (Buchanan and Zakrzewska, 2008).

Based on the course of the symptoms, BMS is classified into three types: type 1- symptoms not presented on waking but arise and increase during the day; type 2- symptoms present all day from waking up; type 3- with symptoms in unusual oral sites and free days of symptoms (van der Waal, 2021).

Another classification, based on the concept that BMS is a neuropathic disorder, identifies two subtypes of this disease: a subgroup characterized by peripheral small diameter fiber neuropathy of the oral mucosa and a subgroup characterized by central neuropathy. In some patients, the presence of both subtypes may occur (Orliaguet and Misery, 2021; van der Waal, 2021).

Despite the fact that BMS is still considered an idiopathic pain disorder, in addition to oral symptoms, psychosocial distress is frequently reported by BMS patients.

The role of psychological factors in BMS has been the focus of several studies, which have shown that the quality of life may be significantly impacted by primary BMS (Pereira et al., 2021), with patients experiencing sleep disturbance (Chainani-Wu et al., 2011), stress (Jedel et al., 2020) depression and anxiety (Buljan et al., 2008; Davies et al., 2015; Sikora et al., 2018; Kim et al., 2020; Malta et al., 2021), and, in some cases, cognitive decline (Canfora et al., 2021).

Although no study could establish a direct causal relationship between BMS and sleep disturbances, several dimensions of sleep have been found altered in patients, including duration, sleep affecting daytime function, sleep quality, efficiency, and the ability to fall asleep (Alhendi et al., 2023). Likewise, the crosstalk between psychological symptoms—mostly depression and anxiety and BMS has not been explained either. However, it has been reported that treatment with different classes of antidepressants, benzodiazepines, as well as cognitive behavior therapy induces benefit in pain score reduction (Tan et al., 2022).

Because of the unclear multifactorial nature of this disorder, a clinical multidisciplinary approach to BMS patients that includes a detailed anamnesis, an oral clinical examination focused on the oral mucosa and any dental problems, a biological evaluation followed by neurological, cardiovascular, and psychiatric evaluations (Aravindhan et al., 2014; Adamo and Spagnuolo, 2022) is recommended.

However, determining the best BMS management strategy remains difficult (McMillan et al., 2016).

The current study’s objectives were to examine the prevalence of stress, depressive and anxiety symptoms, sleep disturbances and cognitive impairments in a large cohort of BMS Romanian patients, compared to a control group of not affected individuals. Furthermore, this study also aimed at investigating, for the first time, the putative presence of semantic and phonemic verbal fluency impairment in BMS patients.

## Materials and methods

A cohort of 120 patients with BMS and 110 non-BMS individuals (CTRL) were enrolled in this study at the Oral Medicine Department, Faculty of Dentistry, “Carol Davila” University of Medicine and Pharmacy. All the BMS patients diagnosed starting from 2017, were recalled for reevaluation in 2022, and those who provided written, informed consent were included in the study. The CTRL group was represented by dental patients (without any BMS symptoms) who were age- and sex-matched with the BMS group.

The study was approved by the scientific research ethics committee of Carol Davila” University (approval number 36988/2022), and all the participants provided written, informed consent. The BMS diagnosis was carried out in accordance with the recommendations of the WHO (World Health Organization, 2023) and the International Headache Society (Headache Classification Committee of the International Headache Society (IHS), 2018). The inclusion criteria for BMS patients were: (i) male or female, aged at least 18 years, (ii) oral symptoms present daily for more than 2 h for at least 3 months, (iii) a normal clinical aspect of the oral mucosa, (iv) a negative oral fungal test, (v) biological tests (complete blood count, serum iron and ferritin, blood sugar levels, B1, B6, and B12 vitamins, as well as the thyroid panel) within a normal range, and (vi) no current psychiatric treatment. The CTRL group inclusion criteria were: (i) male or female, aged at least 18, (ii) absence of BMS symptoms and oral mucosal lesions, (iii) no record of psychiatric illness, and (iv) absence of debilitating medical conditions.

For each participant, the socio-demographic (age, sex, education, BMI, employment, marital status, and use of email, smartphones, and social networks), lifestyle, and habits (tobacco, alcohol, coffee, and fizzy drink consumption, as well as diet type and contact with animals) data were recorded. Each individual was screened by an expert neurologist using standardized questionnaires to assess quality of life with SF-36 scale (score range 0–100) (Ware and Sherbourne, 1992), stress with Perceived Stress Scale - PSS (score range 0–40; 0–13 = low stress, 14–26 = moderate stress, 27–40 = high stress) (Cohen et al., 1983), depression and anxiety symptoms with MADRS (score range 0–60; 0–6 = no depression, 7–19 = mild depression; 20–34 = moderate depression, >35 = severe depression) Williams and Kobak, 2008), and with HADS (score range 0–21; 0–7 = normal, 8–10 = mild; 11–14 = moderate, 15–21 = severe) (Zigmond and Snaith, 1983). The PSQI scale (score range 0–21; >5 = significant sleep disturbance) (Carpenter and Andrykowski, 1998) was used to assess sleep quality. Furthermore, the individuals’ self-reported the hours of night and day sleep durations. Cognitive functions with the MoCA (score range 0–30; <26 = cognitive impairment) (Nasreddine et al., 2005), semantic (SVF), and phonemic verbal fluency (PVF) tests were evaluated in elderly individuals (age > 60 years).

In the SVF the person was asked to list all of the animals he could think of in the next 60 s (cut off: score < 17), and in the PVF test, the person was asked to name all of the words that begin with letter R in 60 s (cut off: score < 17).

Statistical analysis was performed using IBM SPSS v25. All the variables were summed up with descriptive statistics like means, standard deviations, and frequencies. Differences between the groups were assessed using the Chi-squared test and the two-sample t-test for categorical and continuous variables, respectively. Correlations between continuous variables have been performed using Pearson’s test.

## Results

The groups of BMS patients and controls were not different in terms of age, sex, BMI, employment, marital status, and use of email, smartphone and social networks (*p* > 0.05), while a difference in education levels was observed, with BMS patients showing higher average years of schooling than controls (*p* < 0.001). Regarding lifestyle and habits, we found a higher frequency of consumers of coffee (*p* < 0.001), and fizzy drinks (*p* < 0.001) in the BMS group than in the CTRL. Moreover, the BMS patients reported being in more in contact with animals (pets or farm animals) than CTRL (*p* < 0.001) (Table 1).

**Table 1:** Socio-demographic data, lifestyle, and habits of CTRL and BMS patients.

Socio-demographic features			
	CTRL (*N* = 110)	BMS (*N* = 120)	Significance
Age in years (mean ± SD)	59.07 ± 11.43	60.56 ± 11.71	^#^*p* = 0.332
Sex *N* (F%; M%)	82 (74.5%) F; 28 (25.5%) M	93 (77.5%) F; 27 (22.5%) M	^$^*p* = 0.600; *χ*^2^ = 0.275
Education in years (mean ± SD)	13.82 ± 1.04	15.00 ± 1.74	^#^ ***p < 0.001***
BMI (mean ± SD)	26.84 ± 3.13	26.70 ± 3.47	^#^*p* = 0.751
Employment status *N* (%)	Unemployed = 3 (2.7%)	Unemployed = 3 (2.5%)	^$^*p* = 0.970; χ^2^ = 0.537
	Retired = 60 (54.5%)	Retired = 70 (58.3%)	
	Part-time job = 5 (4.5%)	Part-time job = 6 (5%)	
	Full-time job = 31 (28.2%)	Full-time job = 29 (24.2%)	
	Own business = 11 (10%)	Own business = 12 (10%)	
Marital status *N* (%)	Single = 7 (6.4%) Married = 93 (84.5%) Widow/er = 7 (6.4%) Divorced = 3 (2.7%)	Single = 5 (4.2%) Married = 99 (82.5%) Widow/er = 11 (9.1%) Divorced = 5 (4.2%)	^$^*p* = 0.687; χ^2^ = 1.478
e-mail user *N* (%)	No user = 29 (26.4%) Basic user = 39 (35.4%) Advance user = 42 (38.2%)	No user = 37 (30.8%) Basic user = 41 (34.2%) Advance user = 42 (25%)	^$^*p* = 0.746; χ^2^ = 0.586
Smartphone user *N* (%)	No user = 2 (1.9%) Basic user = 48 (43.6%) Advance user = 60 (54.5%)	No user = 6 (5%) Basic user = 60 (50%) Advance user = 54 (45%)	^$^*p* = 0.200; χ^2^ = 3.220
Social Network users *N* (%)	No user = 54 (49.1%) Basic user = 22 (20%) Advance user = 34 (30.9%)	No user = 65 (54.2%) Basic user = 21 (17.5%) Advance user = 34 (28.3%)	^$^*p* = 0.606; χ^2^ = 0.738
Lifestyle and habits			
Tobacco consumers *N* (%)	Non-smoker = 71 (64.5%) Smoker = 39 (35.5%)	Non-smoker = 81 (67.5%) Smoker = 39 (32.5%)	^$^*p* = 0.636; χ^2^ = 0.224
Alcohol consumers *N* (%)	No consumer = 61 (55.5%) Sporadic consumer = 43 (39.0%) Chronic moderate consumer = 6 (5.5%) Severe consumer = 0	No consumer = 81 (67.5%) Sporadic consumer = 35 (29.2%) Chronic moderate consumer = 3 (2.5%) Severe consumer = 1 (0.8%)	^$^*p* = 0.157; χ^2^ = 5.212
Coffee consumers *N* (%)	No consumer = 83 (75.5%) Sporadic consumer = 21 (19.1%) Chronic consumer (1 coffee/day) = 6 (5.4%) Chronic consumer (2–3 coffee/day) = 0	No consumer = 29 (24.2%) Sporadic consumer = 66 (55.0%) Chronic consumer (1 coffee/day) = 23 (19.2%) Chronic consumer (2–3 coffee/day) = 2 (1.6%)	^$^***p* < 0.001; χ**^**2**^ **= 60.958**
Fizzy drinks consumers *N* (%)	No consumer = 97 (88.2%) Sporadic consumer = 12 (10.9%) Chronic consumer (2-3 l/week) = 1 (0.9%)	No consumer = 73 (60.8%) Sporadic consumer = 41 (34.2%) Chronic consumer (2-3 l/week) = 6 (5%)	^$^***p* < 0.001; χ**^**2**^ **= 22.435**
Contact with animals *N* (%)	No animals = 94 (85.4%) Farm animals = 7 (6.4%) Pets = 9 (8.2%)	No animals = 66 (55%) Farm animals = 32 (26.7%) Pets = 22 (18.3%)	^$^***p* < 0.001; χ**^**2**^ **= 25.992**

SD = standard deviation; ^#^*p*-value was calculated with T-test; ^$^*p*-value was calculated with Chi-square test. In bold are reported the significant results.

When assessing the quality of life by the SF-36 scale, we observed that all individuals reported scores indicating from a good to excellent quality of life. However, the score in CTRL was significantly higher than those reported by the patients (*p* < 0.001). The PSS result showed higher levels of stress in BMS patients compared to controls, as well as a higher frequency of individuals with mild and moderate depression as shown by the MADRS (*p* < 0.001) and HADS-D (*p* = 0.001) tests. No difference in anxiety symptoms (HADS-A) was found between the two groups (*p* = 0.174). The analysis of the PSQI test revealed worse sleep quality in BMS patients (*p* < 0.001) than controls. Moreover, a different distribution of sleeping day and night hours was observed between the two groups (p < 0.001) (Table 2).

**Table 2:** Quality of life, stress, depression, anxiety and sleep quality data of CTRL and BMS patients.

Quality of life, stress, depression anxiety and sleep quality			
	CTRL (*N* = 110)	BMS (*N* = 120)	Significance
SF-36 total score (mean ± SD) (min-max)	91.98 ± 4.38 (77–99)	86.10 ± 5.46 (67–95)	^#^ ***p* < 0.001**
PSS sum (mean ± SD)	11.90 ± 4.36	20.93 ± 7.80	^#^ ***p* < 0.001**
MADRS* *N* (%) *Available for 96 CTRL	No depression = 88 (91.7%) Mild depression = 8 (8.3%)	No depression = 72 (60%) Mild depression = 33 (27.5%) Moderate depression = 10 (8.3%) No depression, with treatment = 5 (4.2%)	^$^***p* < 0.001; χ**^**2**^ **= 29.542**
HADS-D *N* (%)	No depression = 109 (99%) Mild Depression = 1 (1%)	No depression = 105 (87.5%) Mild Depression = 15 (12.5%)	^$^***p* = 0.001; χ**^**2**^ **= 11.913**
HADS-A *N* (%)	No Anxiety <8 = 110 (100%)	No Anxiety = 118 (98.3%) Mild Anxiety = 2 (0.7%)	^$^*p* = 0.174; χ^2^ = 1.849
Global PSQI score (min-max)	6.90 ± 4.04 (2–16)	9.62 ± 3.76 (2–18)	^#^ ***p* < 0.001**
Sleeping night hours *N* (%)	≤ 5 h = 0 (0%) 5–6 h = 19 (17.3%) 6–7 h = 80 (72.7%) >7 h = 11 (10%)	≤ 5 h = 5 (4.2%) 5–6 h = 60 (50%) 6–7 h = 53 (44.1%) >7 h = 2 (1.7%)	^$^***p* < 0.001; χ**^**2**^ **= 37.627**
Sleeping day hours (*N* (%))	No day sleep = 97 (88.2%) ≤ 1 h = 11 (10%) 1–2 h = 2 (1.8%)	No day sleep = 72 (60%) ≤ 1 h = 46 (38.3%) 1–2 h = 2 (1.7%)	^$^***p* < 0.001; χ**^**2**^ **= 24.802**

SD = standard deviation; ^#^*p*-value was calculated with T-test; ^$^*p*-value was calculated with Chi-square test. In bold are reported the significant results.

From the entire cohort, we then selected only elderly individuals aged above 60, obtaining the two following sub-groups homogeneous by age: 72 BMS (age mean 68.33 ± 5.84) and 59 CTRL (age mean 67.71 ± 5.04) (*p* = 0.621). These patients and controls underwent a cognitive screening with the MoCA, semantic (SVF), and phonemic verbal fluency (PVF) tests. The case–control analysis did not show any difference in the MoCA score, while a less performance of BMS patients was observed for both the SVF (*p* < 0.001) and PVF (*p* < 0.001) tests (Table 3).

**Table 3:** Cognitive and verbal characteristics of elderly (Age > 60) CTRL and BMS patients.

Cognitive and verbal features			
	CTRL (*N* = 59)	BMS (*N* = 72)	Significance
MoCA (mean ± SD) (min-max)	27.91 ± 1.92 (23–30)	27.69 ± 1.66 (23–30)	^#^*p* = 0.475
SVF (mean ± SD) (min-max)	17.49 ± 1.78 (11–20)	15.46 ± 2.71 (8–21)	^#^ ***p < 0.001***
PVF (mean ± SD) (min-max)	16.02 ± 1.96 (10–19)	12.97 ± 2.65 (6–19)	^#^ ***p < 0.001***

SD = standard deviation; ^#^*p*-value was calculated with T-test; In bold are reported the significant results.

We further correlated, in the BMS elderly patients, the scores of SVF and PVF with those obtained in the tests administered to assess stress, depression, anxiety, sleep quality, and cognitive functions. As expected, SVF and PVF negatively correlated with PSS (*p* = 0.004, Pearson *r* = −0.338; *p* = 0.004, Pearson *r* = −0.337, respectively), with HADS-D (*p* = 0.01, Pearson r = −0.395; *p* = 0.022, Pearson *r* = −0.269, respectively), and with PSQI (p < 0.001, Pearson *r* = −0.486; *p* < 0.001, Pearson *r* = −0.490, respectively). No further correlations were observed between verbal fluency and other parameters.

## Discussion

BMS, a multifactorial condition mostly encountered in women, is a chronic painful disorder that affects the quality of life in most of the patients (Adamo et al., 2019; Pereira et al., 2021). Because its etiology is unclear, a line of research in the last decade has focused on psychological aspects, discovering significant associations between this medical condition and neuropsychological features. In this study, we evaluated a panel of socio-psychological and cognitive features in a large cohort of Romanian BMS patients and not affected individuals in order to identify specific characteristics associated with BMS.

Although the clinicians recommend to BMS patients to avoid acidic foods and liquids, such as tomatoes, orange juice, carbonated beverages, and coffee, analyzing the features related to lifestyle and habits, we observed a higher frequency of fizzy drink intake in BMS patients compared to controls. Moreover, BMS patients reported spending more time with pets and farm animals than not affected individuals. Since it has been demonstrated that interaction with a pet can positively impact quality of life, happiness, and life satisfaction, as well as reduce depressive symptoms (Michalos, 2014), this outcome can represent a tentative way to improve their quality of life, which decreased in BMS patients as reported in our study.

Our study also revealed higher levels of stress in patients than controls measured by the PSS, as reported also by Jedel and collaborators (Jedel et al., 2020) and by Nosratzehi et al. (2020), which, using the Holmes-Rahe questionnaire, found that patients with BMS had more stressful events than controls. Regarding anxiety and depression, in contrast with literature data (Malik et al., 2012; Malta et al., 2021), no anxiety symptoms were registered in the enrolled BMS patients. In our study, the presence of individuals with mild depressive symptoms, evaluated by two different scales, was more frequent in the BMS group than in the control group. The data on depression are in line with those reported in the literature. Indeed, many research groups reported higher levels of depression in BMS patients compared to controls measured by HADS (Malik et al., 2012; Lopez-Jornet et al., 2015; Leuci et al., 2022) and other instruments, including HAM-D (Nasreddine et al., 2005; Canfora et al., 2021, 2023) and the Beck Depression and Anxiety Inventory (BDI) (Buljan et al., 2008; Malta et al., 2021). In addition, in agreement with literature data (Chainani-Wu et al., 2011; Adamo et al., 2013; Lopez-Jornet et al., 2015; Adamo et al., 2018; Rezazadeh et al., 2021), our outcomes confirmed a higher frequency of poor sleep quality in BMS patients than controls. Overall, these findings are consistent with previous research suggesting that circadian rhythm dysfunction, which regulates pain perception, mood, and sleep, may be a clinically significant driver of this disease (Ritchie and Kramer, 2018).

When investigating the presence of signs of cognitive impairment in individuals aged over 60, the MoCA scale did not reveal any statistical difference between patients and controls. Even though the elderly BMS patients had a much higher education level than the controls, they did worse on tests of semantic and phonemic verbal fluency than the controls.

The evaluation of verbal fluency tasks is commonly used for assessing cognitive and linguistic abilities in both healthy and clinical populations (Pekkala, 2012; Thiele et al., 2016; Stielow and Stenneken, 2017; Turkstra, 2018). Both semantic and phonemic fluency evaluations have been used for studying functional brain metabolism in different psychiatric and neurological disorders, such as depression, schizophrenia, bipolar disorder, and other cognitive-linguistic impairments secondary to dementia or head trauma (Opasso et al., 2016; Yeung and Lin, 2021; Tassi et al., 2022). Moreover, verbal fluency scores have shown to be linked with cognitive impairment in mild and moderate Alzheimer’s disease patients (Maseda et al., 2014). In our study, elderly BMS patients did not present general signs of cognitive impairment, according to the MoCA. However, dysfunctions in verbal fluency have been identified. When correlating the semantic and phonemic scores of elderly BMS patients with those obtained in the other neuropsychological tests we found that verbal tasks correlated with perceived stress, depression symptoms, and sleep quality, suggesting that this impairment can be a consequence of the general psychological distress that characterizes BMS patients. This result, to our knowledge, represents a new finding never reported in literature. Indeed, when analyzing the cognitive functions in BMS patients, Canfora et al. found a decline in attention, working memory, and executive functions, but not in praxis-constructive skills or verbal memory (Canfora et al., 2021).

Importantly, this is the first study that has examined verbal fluency in elderly BMS patients without cognitive impairment, finding a correlation between a deficit in verbal skills and BMS that is not related to cognitive decline, our patients being cognitively normal.

Moreover, to our knowledge, this is the largest study in terms of cohort size and number of evaluated features (lifestyle habits, data on quality of life, stress, depression, and anxiety, sleep quality, and cognitive functions) conducted on BMS patients from Eastern Europe.

The limitations of this study are represented by the lack of regression analysis and by the fact that the presence of comorbidities and the associated therapeutic regimen of the enrolled individuals have not been considered. Anyhow, the BMS diagnosis is based on the exclusion of other organic general and oral conditions causing oral pain, and one of the including criteria is the presence of biological tests within normal range.

In conclusion, our data, showing impairments related to quality of life, high levels of stress and depression, as well as sleeping disturbance and verbal fluency impairment, highlight that the BMS condition necessitates specific assistance with attention to psychological, psychiatric, and neurological issues. Taken together, these findings highlight the importance of a multidisciplinary approach to BMS, implying that collaboration between dentists, clinical psychologists, and psychiatrists is required for both diagnosis and treatment.

## Data availability statement

The raw data supporting the conclusions of this article will be made available by the authors, without undue reservation.

## Ethics statement

The studies involving human participants were reviewed and approved by Scientific Research Ethics Committee of the “Carol Davila” University. The patients/participants provided their written informed consent to participate in this study.

## Author contributions

CD designed the study. BP and ST encouraged CD and supervised the project. CD and AR applied the neuropsychological tests. EM, MD, IM, AR, and IP analyzed the data. EM, MD, and IP wrote the manuscript with input from all authors. All authors contributed to the article and approved the submitted version.

## Funding

This work was supported by a grant from the Romanian Ministry of Research, Innovation and Digitization, CCCDI-UUEFISCDI, project number PN-III-P2-2.1-PED-2019-1339 within PNCDI III (contract number 564PED/2021) and 31PFE/30.12.2021.

## Conflict of interest

The authors declare that the research was conducted in the absence of any commercial or financial relationships that could be construed as a potential conflict of interest.

## Publisher’s note

All claims expressed in this article are solely those of the authors and do not necessarily represent those of their affiliated organizations, or those of the publisher, the editors and the reviewers. Any product that may be evaluated in this article, or claim that may be made by its manufacturer, is not guaranteed or endorsed by the publisher.
